# Continuous Indoor Positioning Fusing WiFi, Smartphone Sensors and Landmarks

**DOI:** 10.3390/s16091427

**Published:** 2016-09-05

**Authors:** Zhi-An Deng, Guofeng Wang, Danyang Qin, Zhenyu Na, Yang Cui, Juan Chen

**Affiliations:** 1 School of Information Science and Technology, Dalian Maritime University, Dalian 116026, China; gfwangsh@163.com (G.W.); nazhenyu@dlmu.edu.cn (Z.N.); chenjuan@dlmu.edu.cn (J.C.); 2 School of Electronics Engineering, Heilongjiang University, Harbin 150080, China; qindanyang@hlju.edu.cn; 3 School of Computer Science and Technology, Harbin Institute of Technology, Harbin 150001, China; ycui@hit.edu.cn

**Keywords:** indoor positioning, WiFi, inertial sensor, landmark, extended Kalman filter

## Abstract

To exploit the complementary strengths of WiFi positioning, pedestrian dead reckoning (PDR), and landmarks, we propose a novel fusion approach based on an extended Kalman filter (EKF). For WiFi positioning, unlike previous fusion approaches setting measurement noise parameters empirically, we deploy a kernel density estimation-based model to adaptively measure the related measurement noise statistics. Furthermore, a trusted area of WiFi positioning defined by fusion results of previous step and WiFi signal outlier detection are exploited to reduce computational cost and improve WiFi positioning accuracy. For PDR, we integrate a gyroscope, an accelerometer, and a magnetometer to determine the user heading based on another EKF model. To reduce accumulation error of PDR and enable continuous indoor positioning, not only the positioning results but also the heading estimations are recalibrated by indoor landmarks. Experimental results in a realistic indoor environment show that the proposed fusion approach achieves substantial positioning accuracy improvement than individual positioning approaches including PDR and WiFi positioning.

## 1. Introduction

Indoor location-based service [1] has attracted much attention due to its great social and commercial values, which enable many applications including object finding, human tracking, personalized advertisement, and assisted living, etc. As Global Navigation Satellite Systems (GNSS) signal cannot penetrate well in indoor environment and provide reasonable accuracy performance, various other signals, such as WiFi [2], ultra-wideband [3], Bluetooth [4], radiofrequency identification (RFID) [5], ZigBee [6], and inertial sensors [7] have been developed for indoor positioning. Among them, WiFi positioning using received signal strength (RSS) fingerprinting [8] has been considered as one of the most popular indoor positioning solutions due to its low cost. RSS values from several access points (APs) can be easily gathered by common smartphones under existing WiFi infrastructure. However, severe RSS fluctuations [9] always render inaccurate positioning results.

Another popular indoor positioning solution is pedestrian dead reckoning (PDR) [10] system deploying inertial sensors embedded in a smartphone. PDR determines the user’s location by adding the currently estimated displacement to previously estimated location. The displacement is achieved by combining step detection, step length estimation with user heading estimation using accelerometers, gyroscopes, and magnetometers. PDR using a smartphone is self-contained and requires no additional infrastructures. However, since PDR relies on low cost but noisy inertial sensors, the positioning error may accumulate along walking distances [11].

Both WiFi positioning and PDR approaches have disadvantages and complementary advantages. WiFi positioning approach can provide absolute positioning results to bind the cumulative error drifts of PDR. On the other hand, PDR achieves relatively more accurate positioning over short walking distance, which enables continuous tracking and smoothens the jumping effects of WiFi positioning caused by RSS fluctuations. Additionally, human motion recognition [12] using smartphone sensors may be used to improve indoor positioning. User motion states, like walking stairs, entering and walking out of elevators, and taking escalators, can be treated as indoor landmarks [13], to reset location estimation by PDR. Therefore, recent works tend to fuse WiFi positioning, PDR, and landmarks to enhance the indoor positioning accuracy.

Existing data fusion framework mainly includes particle filter [14,15,16], and Kalman filter [12,17]. The particle filter may achieve reasonable accuracy by deploying a large number of particles [14], but a large amount of computational cost is required and is not suitable for resource limited smartphones. The Kalman filter-based approaches are computational lightweight. However, an explicit measurement equation connecting user’s position with RSS measurements is unavailable due to complex indoor radio propagation, thus rendering the measurement noise statistics unavailable. Previous Kalman filter-based fusion approaches manually and empirically set the related measurement noise covariance matrix. As a result, the fusion process cannot adapt the uncertainty of WiFi positioning results and, thus, rendering a degraded positioning accuracy.

In this paper, a novel extended Kalman filter (EKF)-based approach is deployed to fuse WiFi positioning, PDR, and landmarks. Instead of setting the measurement noise covariance matrix manually, we develop a kernel density estimation (KDE) based measurement model to accurately describe the RSS-position relationship and adaptively measure the related noise statistics. Furthermore, to reduce computational cost and avoid large WiFi positioning errors, we define a trusted area to limit the search space and propose a strategy for detection of outlier RSS measurements, respectively. For PDR, another EKF is designed for user heading estimation by fusing inertial sensors, magnetometers, and landmarks. Unlike previous approaches just deploying landmarks to reset positioning results of PDR, we also exploit the heading estimation information contained in landmarks to recalibrate user heading of PDR.

## 2. Related Works

For WiFi indoor positioning, the most widely used architecture is fingerprinting approach [18]. This approach consists of two phases: offline site survey and online pattern matching. During offline phase, RSS values of several APs are collected at different predefined calibration points to create a fingerprint database named radio map. Then, during online phase, RSS samples are collected in real-time and matched with RSS patterns pre-stored in radio map to derive the user’s location. Due to the complementary advantages of WiFi positioning and PDR, fusing both approaches has been paid increasing attention.

Reference [19] firstly proposes a particle filter fusing inertial signals and WiFi positioning. New particles of user’s location are generated according to the PDR model, once the current user step is identified, the step length and user heading are estimated. Then, the weight of a particle is updated by computing a Gaussian kernel function with variables of particle locations and related WiFi positioning results. Building map information is also exploited to remove the unexpected particles with unreasonable movements. Therefore, the user position is ultimately determined by averaging all weighted particles.

Similarly, [14] also proposes a particle filter fusing low-cost accelerometers, compasses, and WiFi RSS signals. The main difference is the particle weights update scheme, which is achieved by computing the inverse Euclidean distance between real-time observed RSS and mean RSS signal of a particle’s nearest calibration point in the radio map. The particle filter may integrate WiFi positioning, human motion and building map information effectively by deploying a large number of particles. However, it is power consuming and requires extensive computational cost, because location estimation of every walking step requires certain operations on each particle, whose quantity may reach more than several hundreds. Overall, the particle filter-based fusion approach is unsuitable for running on resource limited devices.

Recently, [12] proposes a light computation approach by deploying Kalman filter to fuse WiFi positioning, PDR, and landmarks. This approach formulates PDR as a linear function with a known user heading and step length. Consequently, inaccurate estimation of the error covariance of the state vector may be achieved due to the nonlinear nature of PDR versus the variables, including user heading and step length, thus rendering degraded positioning accuracy performance of the Kalman filter. For WiFi positioning, the user position is estimated by averaging the weighted coordinates of APs. The related weights are inversely proportional to the physical distances between smartphones and APs, which are computed by constructing and exploiting the RSS signal propagation model. Though a radio map is not required by WiFi positioning, due to the complexity and uncertainty of the indoor wireless propagation environment [18], the indoor signal propagation model may be so inaccurate that positioning results with large errors will be likely introduced to the Kalman filter. Furthermore, the measurement noise variance is set empirically, which cannot adapt the uncertainty of WiFi positioning results. More recently, Xin Li et al. [17] develop an EKF to fuse WiFi fingerprinting positioning with PDR. Similarly to [12], the measurement noise variance matrix is empirically set as a constant value, thus rendering degraded positioning accuracy of EKF.

We propose an EKF-based fusion approach with lightweight computation. For WiFi positioning, due to complex wireless propagation factors, such as non-line-of-sight propagation, it is difficult to develop an explicit measurement equation for WiFi RSS measurements [20]. Therefore, we develop a KDE-based [21,22] measurement model, which may adaptively measure uncertainty of WiFi positioning. To reduce the computational cost, we restrict the WiFi positioning search space into a trusted area defined by fusion position results of the previous step. Additionally, to avoid introducing large positioning errors into the EKF fusion process, we propose a detection algorithm to filter out WiFi signal outliers.

For heading estimation of PDR, rather than deploy Euler angles [23] and a direction cosine matrix (DCM) [24], we deploy a quaternion [7,25] because it can avoid the singularity problems and consume much less computational resources than DCM. Furthermore, to compensate the heading estimation drift, we design another EKF to fuse the three dimensional gyroscopes, accelerometers, and magnetometers. To reduce accumulation error of PDR, not only positioning results are reset by landmarks, but also the user heading estimation may be recalibrated, since the landmarks always indicate a specific region of user heading.

## 3. Methodology

**Figure 1:**
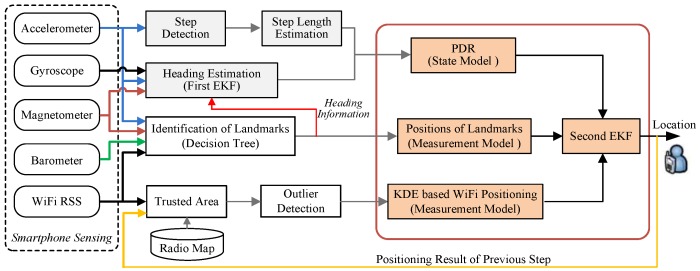
Overview of the proposed extended Kalman filter (EKF)-based fusion approach.

Figure 1 overviews the proposed fusion approach for indoor positioning using smartphones. The proposed approach consists of two EKFs. The first one is used for heading estimation of PDR to integrate gyroscopes, accelerometers, magnetometers, and landmarks. The second one estimates the user’s location by fusing WiFi positioning, PDR, and landmarks.

For the second EKF, the measurement model includes the KDE based WiFi positioning algorithm and landmarks for position resets. For WiFi positioning, we exploit the positioning results of previous step to adaptively restrict search space of WiFi positioning into a trusted area, and develop a strategy for detection of outlier RSS measurements. The state model is PDR system, which consists of step detection, step length estimation, and quaternion-based heading estimation modules. For heading estimation of PDR, it is also recalibrated by exploiting user heading information contained in landmarks. The landmarks can be identified by a decision tree using accelerometers, barometers, magnetometers, and WiFi RSS signals. For convenience, we provide a list of key symbols used in the proposed fusion approach in Table 1.

### 3.1. WiFi Positioning

#### 3.1.1. Kernel Density Estimation Based Measurement Update

This section describes the KDE-based WiFi positioning for constructing measurement model of the second EKF. Due to the complex propagation environment [26], such as non-line-of-sight propagation and human body absorptions, an explicit measurement equation relating user position to RSS measurements and related measurement noise statistics are unknown. Instead, fingerprinting approach collects RSS samples at a predefined set of calibration points to implicitly construct the RSS position relationship. Previous Kalman filter-based approaches always set the measurement noise covariance matrix empirically through a rough analysis of the positioning error. Consequently, an inaccurate estimation of measurement noise statistics will degrade the performance of Kalman filter.

Though measurement noise statistics of WiFi positioning is unavailable, we can approximately obtain it if we know the posterior density of the user’s location with related RSS vector samples. Assume that r is the RSS vector sample collected in real-time, l is the user’s two-dimensional coordinate vector, and the posterior density of the user’s location is f(l|r). We give the location estimation and its covariance matrix based on Minimum Mean Squared Error (MMSE) [27] criteria:

(1) $$\hat{l}=\int lf\left(l|r\right)dr$$

(2) $$P_{r}=\int \left(l-\hat{l}\right){\left(l-\hat{l}\right)}^{T}f\left(l|r\right)dr$$

To obtain the posterior density, we deploy kernel density estimation (KDE), a nonparametric density estimator, due to its successful applications in probability density distributions estimation [21]. Given a set of RSS training pairs $\left\{\left({\bar{r}}_{i},l_{i}\right)|i=1,\ldots ,N\right\}$ stored in the radio map with mean RSS vector ${\bar{r}}_{i}$ at the calibration point $l_{i}$, and deploying the widely used Gaussian kernel function ℘, the posterior density estimation can be rewritten as follows:

(3) $$\hat{f}\left(l|r\right)=\frac{\hat{f}\left(l,r\right)}{\hat{f}\left(r\right)}=\frac{\sum_{i=1}^{N}℘\left(r;{\bar{r}}_{i},\Sigma_{r}\right)℘\left(l;l_{i},\Sigma_{l}\right)}{\sum_{i=1}^{N}℘\left(r;{\bar{r}}_{i},\Sigma_{r}\right)}=\sum_{i=1}^{N}{\tilde{w}}_{i}\left(r\right)℘\left(l;l_{i},\Sigma_{l}\right)$$

(4) $${\tilde{w}}_{i}\left(r\right)=\frac{℘\left(r;{\bar{r}}_{i},\Sigma_{r}\right)}{\sum_{i=1}^{N}℘\left(r;{\bar{r}}_{i},\Sigma_{r}\right)}$$

(5) $$℘\left(r;{\bar{r}}_{i},\Sigma_{r}\right)={\left(2\pi \right)}^{-d/2}{\left|\Sigma_{r}\right|}^{-1/2}\exp\left\{-\frac{1}{2}{\left(r-{\bar{r}}_{i}\right)}^{T}\Sigma_{r}\left(r-{\bar{r}}_{i}\right)\right\}$$

(6) $$℘\left(l;l_{i},\Sigma_{l}\right)={\left(2\pi \right)}^{-1}{\left|\Sigma_{l}\right|}^{-1/2}\exp\left\{-\frac{1}{2}{\left(l-l_{i}\right)}^{T}\Sigma_{l}\left(l-l_{i}\right)\right\}$$

where N is the number of calibration points at the target positioning environment, d is the number of APs used in RSS vector, ${\bar{r}}_{i}$ and $l_{i}$ are mean vector of the Gaussian kernel functions, and diagonal matrices $\Sigma_{r}$ and $\Sigma_{l}$ are the corresponding covariance matrices of the Gaussian kernel functions, respectively. To obtain the parameter $\Sigma_{r}$, we use the estimation method given by Silverman [22],

(7) $$\Sigma_{r}=\sigma^{*}I_{d}$$

(8) $$\sigma^{*}={\left(\left(2d+1\right)n/4\right)}^{-1/\left(d+4\right)}\tilde{\sigma }$$

where $I_{d}$ is d×d identity matrix, $\tilde{\sigma }=1/d\sum_{i=1}^{d}\sigma_{i}^{2}$ is the averaged value of marginal variances for RSS values of each AP. Such an estimation of the kernel parameter has been shown to be effective in WiFi positioning [26] and can be also verified in our experimental results. For the parameter $\Sigma_{l}$ of Gaussian kernel, it represents the walking velocity of pedestrians and can be set as the classical pedestrian velocity values.

As seen in Equation (3), the posterior distribution can be considered as a Gaussian mixture of $℘\left(l;l_{i},\Sigma_{l}\right),\;i=1,\ldots ,N$ with the weighting value ${\tilde{w}}_{i}\left(r\right)$ for each calibration point. Therefore, the MMSE location estimation and its error covariance using RSS measurements only may be determined by the first two moments [27] as follows:

(9) $$\hat{l}=\sum_{i=1}^{N}{\tilde{w}}_{i}\left(r\right)l_{i}$$

(10) $$P_{r}=\sum_{i=1}^{N}{\tilde{w}}_{i}\left(r\right)\left(\Sigma_{l}+\left(l_{i}-\hat{l}\right){\left(l_{i}-\hat{l}\right)}^{T}\right)$$

Equations (9) and (10) can be used for WiFi positioning and the corresponding covariance matrix used in the measurement update step of the second EKF, respectively.

#### 3.1.2. Trusted Area for Calibration Points Selection

For KDE-based WiFi positioning, the location estimation in Equation (9) is a weighted average of calibration points, where the weight is inversely proportional to the distance between the RSS vectors collected in real-time and the fingerprints pre-stored in the radio map. It may incur extensive computational cost if the real-time collected RSS vector is matched with the whole radio map consisting of a large number of calibration points. Furthermore, due to RSS fluctuations, some calibration points far away from the user’s true position may receive relatively high weights and degrades the WiFi positioning accuracy.

To address these problems, we define a trusted area to restrict the WiFi positioning into a small set of the calibration points instead of the whole radio map. The basic idea for defining a trusted area is that the physical distance between adjacent walking steps is rather limited during pedestrian walking. Thus, we define the center of the trusted area by the positioning output of previous step and its size by location estimation covariance of the fusion approach. Define that ${\hat{L}}_{\mathrm{Previous}}$ is the positioning result of the proposed approach of previous walking step, $D_{\mathrm{Th}}$ is the radius of the trusted area. The calibration points involved in KDE-based WiFi positioning for the current step can be selected:

(11) $$S_{\mathrm{Current}}=\left\{\left({\bar{r}}_{i},l_{i}\right)|\;{\left\|l_{i}-{\hat{L}}_{\mathrm{Previous}}\right\|}_{2}\leq D_{\mathrm{Th}};i=1,\ldots ,N\right\}$$

where the radius of the trusted area $D_{\mathrm{Th}}$ is determined by location estimation covariance of the previous walking step, which may be set appropriately by controlling the probability that the error vector lies in the trusted area reaching 98.89% [28]. This 98.89% is just the probability that the variable lies within the three-sigma point if the variable is assumed to be Gaussian distributed. The cardinality of the set $S_{\mathrm{Current}}$ is denoted as $N_{\mathrm{Current}}$, which is always much smaller than the total number of calibration points *N*. Therefore, the defined trusted area greatly reduces the number of calibration points involved and, thus, reduces the computational cost of WiFi positioning. This strategy also avoids deploying a clustering analysis technique [29,30], which is used in many previous works to reduce computational cost.

#### 3.1.3. Outlier Detection of RSS Measurements

For EKF-based fusion approach, an erroneous location estimation caused by outlier of real-time RSS measurements can propagate to future location estimations, degrading positioning accuracy. To avoid introducing large positioning errors caused by RSS measurements outliers, we propose an outlier detection algorithm. We use the averaged weight of the calibration points in the trusted area as an indication of RSS reliability:

(12) $$\eta \left(r\right)=\sum_{i=1}^{N_{\mathrm{Current}}}w_{i}\left(r\right)/N_{\mathrm{Current}}=\sum_{i=1}^{N_{\mathrm{Current}}}℘\left(r;{\bar{r}}_{i},\Sigma_{r}\right)/N_{\mathrm{Current}}$$

where the averaged weight is bounded and $0<\eta \left(r\right)<{\left(2\pi \right)}^{-d/2}{\left|\Sigma_{r}\right|}^{-1/2}$ [21], d is the dimensionality of related RSS vectors. A small value of the averaged weight indicates that the real-time RSS measurement does not match most of the fingerprints in the trusted area and may, therefore, be considered as an outlier. The threshold value of the averaged weight may be set by statistical analysis of the averaged weights of normal RSS measurements during offline phase. Once an outlier is detected, the measurement update from WiFi positioning is omitted and a large positioning error introduced into the fusion process is avoided.

### 3.2. Identification of Landmarks

**Figure 2:**
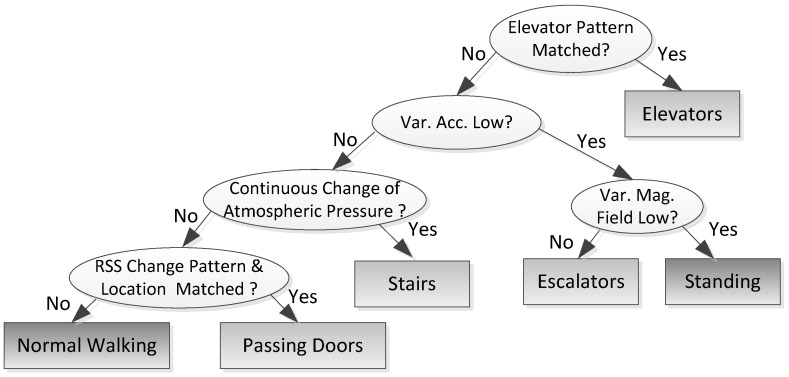
Identification of landmarks using a decision tree.

Previous works have shown that the landmarks may be used to reset location estimation of PDR. Particularly, landmarks may accurately restart location estimation of PDR once pedestrians reach these landmarks. The landmarks explored in this paper consist of elevators, escalators, stairs, and doors. When users walk into or out of elevators, take escalators, walk upstairs/downstairs, or pass through a door when entering or leaving a room, the user headings are usually restricted into a small specific range. Therefore, we further explore landmarks to recalibrate the heading estimation. We deploy a decision tree including four levels to detect these landmarks and separate them from normal walking and standing motion states, as shown in Figure 2. To exploit the landmarks, accurate mapping of the whole indoor environment, such as the size of the rooms and their relative locations, is not required, while knowing the locations of these landmarks is enough.

In the first level, we firstly identify the elevator landmarks based on their unique pattern of acceleration signals [13]. A typical process of taking an elevator consists of normal walking, followed by standing sometime to wait for the elevator, entering it, standing inside for a short period, a pair of hyper-gravity/hypo-gravity effect with positive/negative impulses of vertical acceleration, a stationary period between two impulses depending on the elevation variations of the elevator, and finally a walk-out. In order to exploit the user heading information, we also deploy the magnitude of the magnetic field to accurately capture the epoch of walking into or out of an elevator. A significant decrease or increase of the total magnitude of magnetic field occurs when a pedestrian walks into or out of an elevator.

In the second level, we exploit the acceleration variances to distinguish between taking escalators/standing and walking stairs/walking, since the walking stairs/walking states involve the higher locomotion intensity and, consequently, the larger acceleration variances than those of taking escalators/standing. In the third level, we exploit the variances of the magnetic field values to separate escalators from standing, since the magnetic field values vary rapidly with the motion of escalators.

To further distinguish between walking and walking stairs, we deploy a barometer, whose atmospheric pressure value decreases with the increasing elevation [12]. We compute the absolute difference values of neighboring samples, and deploy a sum of the values within a sliding window as a flag. If the flag exceeds a threshold, walking upstairs/downstairs are identified. To reduce the influence of the sampling noise, we generate one sample by averaging all raw samples collected within one second. Though the barometer may indicate the variations in altitude and help three-dimensional positioning, we just focus two-dimensional positioning in this paper, and only exploit the barometer for landmark identification. We will expand to three-dimensional positioning in our future work.

In the fourth level, we deploy the RSS change pattern and location of the pedestrian to identify the landmarks of doors. For RSS values, it is well known that passing through doors may render large changes of RSS values from multiple APs due to the present or absent of signal attenuations of walls. Furthermore, to avoid confusion between different doors and false positive events, we also compare the current pedestrian location with the true location of doors to determine whether there is a door nearby.

Note that uncertainties of heading estimation information obtained from different landmarks are different. For example, user heading of taking an escalator has a smaller variation than that of walking upstairs/downstairs. Practically, measurement noises of user headings and user positions from different landmarks may be set according to realistic environments. Through identification of landmarks based on the decision tree approach, the average classification accuracy of landmarks can reach as high as 99%. We collected 200 test samples for each motion state (landmark) and a total number of 1200 test samples in our indoor office environment, except for escalator samples collected in a shopping mall environment. The confusion matrix for the identification of different landmarks is shown in Table 2. Table 2 shows that nearly all landmarks can be identified correctly, except for the negligible confusion between walking and stairs, walking and passing doors, due to the variations of atmospheric pressure and magnetic field values, respectively. Thus, we assume that all of the landmarks can be correctly identified in the proposed fusion approach.

### 3.3. Pedestrian Dead Reckoning (PDR)

PDR determines the position of current step by adding displacement to that of a previous step,

(13) $$L_{i}\;=\;L_{i-1}\;+\;SL_{i}\left[\begin{array}{c} \;\cos({\tilde{\psi }}_{i})\\ \sin({\tilde{\psi }}_{i}) \end{array}\right]$$

where $L_{i}\;=\;\left(x_{i},y_{i}\right)$ and $L_{i-1}\;=\;\left(x_{i-1},y_{i-1}\right)$ are the two-dimensional coordinate vectors of current (the *i*-th) and the related previous steps, $SL_{i}$ is the current step length, and ${\tilde{\psi }}_{i}$ is the user heading. In this paper, the initial position and user heading of PDR are assumed to be known, which can be obtained by various methods [31,32], such as the user’s input and the landmarks. For example, when a pedestrian walks out of an elevator, we can reset the position and user heading of PDR.

#### 3.3.1. Step Detection and Step Length Estimation

We deploy the widely used windowed peak detection algorithm [7] to recognize each walking step. The step detection relies on periodic acceleration fluctuation patterns, whose peak points associate with the heel strike during walking. The total magnitude of acceleration vector is used as an input of the peak detection algorithm, which is insensitive to device orientations and more robust than the widely used vertical acceleration values. Furthermore, coordinate transformation process for the acceleration signal is also not required during the pedestrian step detection. To reduce the signal noise, we average the raw acceleration vector sample by a centered sliding window. Then, the peak detection algorithm is applied to detect every distinct walking step. To avoid the false peaks caused by acceleration jitters, we use two thresholds to limit the magnitude of peaks and the time interval between neighboring peaks:

(14) $$\{\begin{array}{l} \left|AccNorm-g\right|\geq A_{\mathrm{Th}}\\ \Delta T\geq T_{\mathrm{Th}} \end{array}$$

where AccNorm is the total magnitude of acceleration vector, g is the local gravity value, $A_{\mathrm{Th}}$ represents the magnitude threshold of peaks, ΔT is the time interval between neighboring peaks, $T_{\mathrm{Th}}$ represents the shortest time interval allowed between neighboring steps. In fact, we set the time threshold around two-thirds of the duration of one step. For the false peak of a small magnitude, it will be filtered out by the magnitude threshold. For the false peak with magnitude exceeding the magnitude threshold, it can also be effectively filtered out, since the time interval between the false peak and the true one within the same step will be much shorter than the time threshold.

Various linear or nonlinear step length estimation models [33] have been developed. These models relate estimated step length to variables such as step frequency and pedestrian height. In fact, it is not an easy task to construct a generic step length estimation model to adapt different users with different environments. We deploy the following linear estimation model:

(15) StepLength SL =α⋅fre+β⋅var+γ

where fre is step frequency; var is variance of accelerations within one step; α and β are the related coefficients; γ is the step constant. These parameters can be accurately estimated by offline training.

#### 3.3.2. First EKF: User Heading Estimation Fusing Inertial Sensors, Magnetometers, and Landmarks

**Figure 3:**
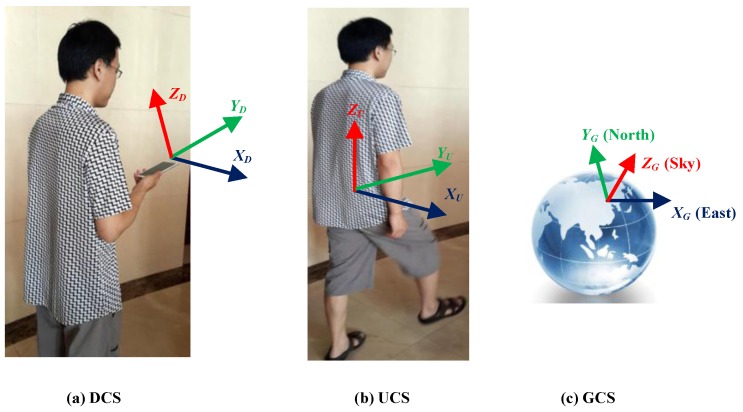
Definitions of three coordinate systems: (**a**) device coordinate system (DCS); (**b**) user coordinate system (UCS); and (**c**) global coordinate system (GCS).

The state model of the first EKF deploys quaternion to describe device orientation time-evolutions, since the singularity problems can be avoided [25]. The measurement model of the first EKF includes an accelerometer for calibrating pitch and roll angles of the device, a magnetometer and landmarks for calibrating the yaw angle of the device. For user heading estimation, we define three coordinate systems, including device coordinate system (DCS), user coordinate system (UCS), and global coordinate system (GCS), as seen in Figure 3. As seen in Figure 3a, DCS is defined by axes $X_{DCS}$, $Y_{DCS}$, and $Z_{DCS}$ $X_{DCS}$ and $Y_{DCS}$ axes point to right and forward, respectively, and both of them are parallel to the front face of the device screen; the $Z_{DCS}$ axis is perpendicular to $X_{DCS}$ and $Y_{DCS}$ axes and points outside of screen. All sensor signals consisting of three-dimensional angular velocities, accelerations, and magnetic field are collected at DCS. As seen in Figure 3b, The UCS is defined by axes $X_{U}$, $Y_{U}$, and $Z_{U}$, where $Y_{U}$ is tangential to the walking trajectory, $Z_{U}$ is the opposite direction of the gravity vector, and $X_{U}$ is the cross product of $Y_{U}$ and $Z_{U}$. As seen in Figure 3c, The GCS is the Earth coordinate system defined by axes $X_{G}$, $Y_{G}$, and $Z_{G}$, which point east, north, and coincide with $Z_{U}$, respectively. User heading estimation can be considered as finding the relative orientation of UCS corresponding to the GCS. The user heading angle is defined as the anticlockwise rotation angle from the real-north direction $Y_{G}$ to walking direction $Y_{U}$. In this paper, as in many other works [12,17], we only consider one of the most common device carrying positions: hand-held position, as seen in Figure 3a. The horizontal component of device forward axis ($Y_{D}$) coincides with walking direction $Y_{U}$. Therefore, user heading is determined if DCS is specified corresponding to GCS by deploying rotation quaternion. Besides, for smartphone placed in the other positions, such as in the trouser pocket, principal component analysis can be used to extract the walking direction. Reference [34], which is our previous preliminary work in that research direction, is recommended.

Firstly, we connect user heading with a rotation quaternion vector by transforming a 3×1 column-vector from GCS to DCS as follows:

(16) $$h^{DCS}\left(t\right)=C_{GCS}^{DCS}\left(q\left(t\right)\right)h^{GCS}\left(t\right)$$

where $C_{GCS}^{DCS}\left(q\left(t\right)\right)$ is the direction cosine matrix (DCM); $h^{DCS}\left(t\right)$ and $h^{GCS}\left(t\right)$ are the column-vector at DCS and GCS; $q\left(t\right)=\;{\left[\begin{array}{cccc} q_{0} & q_{1} & q_{2} & q_{3} \end{array}\right]}^{T}$ is the normalized orientation quaternion with the scalar part $q_{0}$ and the vector part $q_{1},q_{2},q_{3}$. For simplicity, we omit the argument of time t. DCM can be described by the quaternion:

(17) $$C_{GCS}^{DCS}\left(q\right)=\;\left[\begin{array}{ccc} q_{0}^{2}+q_{1}^{2}-q_{2}^{2}-q_{3}^{2} & 2\left(q_{1}q_{2}+q_{0}q_{3}\right) & 2\left(q_{1}q_{3}-q_{0}q_{2}\right)\\ 2\left(q_{1}q_{2}-q_{0}q_{3}\right) & q_{0}^{2}-q_{1}^{2}+q_{2}^{2}-q_{3}^{2} & 2\left(q_{0}q_{1}+q_{2}q_{3}\right)\\ 2\left(q_{1}q_{3}+q_{0}q_{2}\right) & 2\left(q_{2}q_{3}-q_{0}q_{1}\right) & q_{0}^{2}-q_{1}^{2}-q_{2}^{2}+q_{3}^{2} \end{array}\right]$$

The yaw angle of a smartphone at GCS is given as a function of quaternion vector f(q):

(18) $$yaw=f\left(q\right)=\{\begin{array}{cc} \arcsin\left(2\left(q_{0}q_{3}-q_{1}q_{2}\right)/\sqrt{1-4{\left(q_{0}q_{1}+q_{2}q_{3}\right)}^{2}}\right), & q_{0}^{2}-q_{1}^{2}+q_{2}^{2}-q_{3}^{2}>0\\ \pi -\arcsin\left(2\left(q_{0}q_{3}-q_{1}q_{2}\right)/\sqrt{1-4{\left(q_{0}q_{1}+q_{2}q_{3}\right)}^{2}}\right), & \mathrm{otherwise} \end{array}$$

Secondly, according to the kinematic law of rigid body [25], the discrete time evolution model of orientation quaternions is:

(19) $$q_{k+1}\;=\;\exp\left(0.5\times \Omega \left(w_{k}T_{s}\right)\right)q_{k}=\left(I_{4}\cos\left(0.5\times \Delta \theta_{k}\right)+\Omega \left(w_{k}T_{s}\right)\sin\left(0.5\times \Delta \theta_{k}\right)/\Delta \theta_{k}\right)q_{k}$$

where $q_{k}$ and $q_{k+1}$ are the quaternions at time instants $kT_{s}$ and $\left(k+1\right)T_{s}$, $T_{s}$ is the system interval, $w_{k}={\left[\begin{array}{ccc} w_{k}^{x} & w_{k}^{y} & w_{k}^{z} \end{array}\right]}^{T}$ is the angular velocity vector measured at DCS with time instants $kT_{s}$, $I_{4}$ is 4×4 identity matrix, $\Delta \theta_{k}=T_{s}\sqrt{{\left(w_{k}^{x}\right)}^{2}+{\left(w_{k}^{y}\right)}^{2}+{\left(w_{k}^{z}\right)}^{2}}$, $\Omega \left(w_{k}T_{s}\right)$ is given as follows:

(20) $$\Omega \left(w_{k}T_{s}\right)=T_{s}\left[\begin{array}{cccc} 0 & -w_{k}^{x} & -w_{k}^{y} & -w_{k}^{z}\\ w_{k}^{x} & 0 & w_{k}^{z} & -w_{k}^{y}\\ w_{k}^{y} & -w_{k}^{z} & 0 & w_{k}^{x}\\ w_{k}^{z} & w_{k}^{y} & -w_{k}^{x} & 0 \end{array}\right]$$

Therefore, the quaternion $q_{k+1}$ is determined, starting from initial condition $q_{0}$, which can be easily calculated by initial known device orientation at GCS.

Finally, we give the state and measurement model of the first EKF for user heading estimation. We deploy the related device quaternions as the state vector and, thus, the state model can be given as:

(21) $$q_{k+1}\;=\;F_{k}q_{k}+w_{k}^{q}$$

where state transition matrix $F_{k}=\exp\left(\Omega \left(w_{k}T_{s}\right)\right)$, and:

(22) $$w_{k}^{q}=\Xi_{k}w_{k}^{gyro}=-\frac{T_{s}}{2}\left[\begin{array}{c} {}[e_{k}\times ]+q_{0}^{k}I_{3}\\ -e_{k}^{T} \end{array}\right]w_{k}^{gyro}$$

where $q_{k}=\;{\left[\begin{array}{cccc} q_{0}^{k} & q_{1}^{k} & q_{2}^{k} & q_{3}^{k} \end{array}\right]}^{T}$includes the scalar part $q_{0}^{k}$ and the vector part $e_{k}=\;{\left[\begin{array}{ccc} q_{1}^{k} & q_{2}^{k} & q_{3}^{k} \end{array}\right]}^{T}$, $I_{3}$ is 3×3 identity matrix, $w_{k}^{gyro}$ is the white Gaussian noise [25] of gyroscope outputs at time instants $kT_{s}$, and $\left[e_{k}\times \right]$ is a standard vector cross-product operator. To obtain the zero-bias offset and scaling factor of gyroscope, we deploy the widely used calibration method called zero attitude update [35], which is implemented at the initial time of each experimental run. Equations (21) and (22) describing the time evolution of quaternion can be considered as a first order approximation of the exact model Equation (19). The measurement noise vector of gyroscope $w_{k}^{gyro}$ is assumed small enough after calibration process, thus a first order approximation always performs well. The process noise covariance matrix $Q_{k}$ can be given as:

(23) $$Q_{k}\;=\;\Xi_{k}Q_{k}^{gyro}\Xi_{k}^{T}$$

The measurement model of the first EKF is generated by relating real-time measured acceleration vectors at DCS, real-time magnetic field vectors at DCS to the local gravity and magnetic field vectors at GCS using the DCM as seen in Equation (16). The user heading derived from landmarks are also added in the measurement model.

(24) $$z_{k+1}=\left[\begin{array}{c} a_{k+1}\\ m_{k+1}\\ yaw_{k+1} \end{array}\right]=\phi \left(q_{k+1}\right)+v_{k+1}=\left[\begin{array}{ccc} C_{GCS}^{DCS}\left(q_{k+1}\right) & 0 & 0\\ 0 & C_{GCS}^{DCS}\left(q_{k+1}\right) & 0\\ 0 & 0 & 1 \end{array}\right]\left[\begin{array}{l} g\\ h\\ f\left(q_{k+1}\right) \end{array}\right]+\left[\begin{array}{c} v_{k+1}^{a}\\ v_{k+1}^{m}\\ v_{k+1}^{yaw} \end{array}\right]$$

where $a_{k+1}$ and $m_{k+1}$ are the acceleration and magnetic field vectors of the smartphone measured at DCS, g and h are the local gravity and magnetic field vectors at GCS, $v_{k+1}^{a}$ and $v_{k+1}^{m}$ are the white Gaussian measurement noise vectors of the accelerometer and gyroscope; $f\left(q_{k+1}\right)$ is the yaw angle at GCS derived from the quaternion vector as seen in Equation (18); $yaw_{k+1}$ is the yaw angle derived from a landmark and $v_{k+1}^{yaw}$ is the related measurement noise. The covariance matrix of the measurement model $R_{k+1}$ can be given as follows:

(25) Rk+1=[Rk+1a000Rk+1m000Rk+1yaw]=[σa2RI3000σm2RI3000σyaw2R]

To filter out the acceleration perturbations and severely perturbed magnetic field values, we adaptively tune the parameters of measurement covariance matrix for observed signals:

(26) σa2R={σa2,‖ak+1−g‖2<εa1 ∩var(‖ak+1−Na/2‖2:‖ak+1+Na/2‖2)<εa2∞,otherwise

(27) σm2R={σm2,‖mk+1−h‖2<εm1 ∩var(‖mk+1−Nm/2‖2:‖mk+1+Nm/2‖2)<εm2∞,otherwise

where $\varepsilon_{a1}$ and $\varepsilon_{m1}$ represent the allowed maximum deviations of acceleration vector and magnetic field vector from the local gravity vector and the local magnetic field vector, respectively; $\varepsilon_{a2}$ and $\varepsilon_{m2}$ represent the corresponding allowed maximum variances, respectively; $N_{a}$ and $N_{m}$ define the number of acceleration and magnetic field samples involved in computing the variances of acceleration and magnetic field signals, respectively; var(⋅) is the variance of windowed signals. For observed yaw angle derived from a landmark, the measurement noise variance σyaw2R is set during offline phase according to the style of a landmark and related realistic environments, as described in Section 3.2.

To obtain the linearized version of (24), the EKF approach linearizes the nonlinear function ϕ(⋅) of Equation (24) by computing the related Jacobian matrix:

(28) $$H_{k+1}={\frac{\partial }{\partial q_{k+1}}\phi \left(q_{k+1}\right)|}_{q_{k+1}=q_{k+1}^{-}}$$

where the true values of state $q_{k+1}$ are unavailable. We replace $q_{k+1}$ by the best estimation available, called the a priori state estimation $q_{k+1}^{-}$.

Based on the state model in Equation (21), the linearized version of measurement model in Equation (24), and related process and measurement noise covariance matrices, the a *posteriori* estimation of the quaternion ${\hat{q}}_{k+1}$ and the related covariance matrix $P_{q}^{k+1}$ can be obtained by typical procedure of the EKF. We briefly summarize the detailed procedures as follows:

**Predicting:**

(29) $$q_{k+1}^{-}=F_{k}{\hat{q}}_{k}$$

(30) $$P_{q-}^{k+1}=F_{k}P_{q}^{k}F_{k}^{T}+Q_{k}$$

**Updating:**

(31) $$K_{k+1}=P_{q-}^{k+1}H_{k+1}^{T}{\left(H_{k+1}P_{q-}^{k+1}H_{k+1}^{T}+R_{k+1}\right)}^{-1}$$

(32) $${\hat{q}}_{k+1}=q_{k+1}^{-}+K_{k+1}\left(z_{k+1}-\phi \left(q_{k+1}^{-}\right)\right)$$

(33) $$P_{q}^{k+1}=P_{q-}^{k+1}-K_{k+1}H_{k+1}P_{q-}^{k+1}$$

After obtaining ${\hat{q}}_{k+1}$, the user heading can be ultimately determined as in Equation (18).

### 3.4. Second EKF: Fusing WiFi Positioning, PDR, and Landmarks

This section describes the second EKF based fusion approach, which exploits complementary strengths of WiFi positioning, PDR, and landmarks. We deploy KDE-based WiFi positioning and landmarks for measurement updates, and deploy PDR for the state model.

Firstly, we construct the state model, whose state vector is the user’s two-dimensional coordinate vector. For the target indoor environment, a specific predefined environment coordinate system may be given according to building structures or personalized requirements. Assume $\psi_{i\_step}$ is the yaw angle estimated at the ith step from Equation (18) of the first EKF at GCS, and $\psi_{x\_\text{axis}}$ is the yaw angle of the positive *x*-axis of the environment coordinate system. Therefore, the anticlockwise rotation angle from the positive *x*-axis of a predefined environment coordinate system to the walking direction at the ith step, which is denoted as ${\tilde{\psi }}_{i}$, can be given as ${\tilde{\psi }}_{i}=\psi_{i\_step}-\psi_{x\_\text{axis}}$. Then, PDR model can be rewritten by substituting ${\tilde{\psi }}_{i}=\psi_{i\_step}-\psi_{x\_\text{axis}}$ into Equation (13):

(34) $$L_{i}=\;L_{i-1}\;+\;\tilde{f}\left(qd_{i}\right)=L_{i-1}\;+\;SL_{i}\left[\begin{array}{c} {\;\cos(\psi }_{i\_step}{)\cos(\psi }_{x\_\text{axis}}{)+\sin(\psi }_{i\_step}{)\sin(\psi }_{x\_\text{axis}})\\ {\sin(\psi }_{i\_step}{)\cos(\psi }_{x\_\text{axis}})-{\sin(\psi }_{x\_\text{axis}}{)\cos(\psi }_{i\_step}) \end{array}\right]$$

(35) $$\left\{ \begin{array}{l} \cos\left(\psi_{i\_step}\right)=\left(1-2{\left(q_{1}^{i\_step}\right)}^{2}-2{\left(q_{3}^{i\_step}\right)}^{2}\right){\left(1-4{\left(q_{0}^{i\_step}q_{1}^{i\_step}+q_{2}^{i\_step}q_{3}^{i\_step}\right)}^{2}\right)}^{-0.5}\\ \sin\left(\psi_{i\_step}\right)=\left(2q_{0}^{i\_step}q_{3}^{i\_step}-2q_{1}^{i\_step}q_{2}^{i\_step}\right){\left(1-4{\left(q_{0}^{i\_step}q_{1}^{i\_step}+q_{2}^{i\_step}q_{3}^{i\_step}\right)}^{2}\right)}^{-0.5} \end{array}\right.$$

where $\tilde{f}\left(\cdot \right)$ is the relative displacement function of variable $qd_{i}={\left[\begin{array}{cc} q_{i\_step}^{T} & SL_{i} \end{array}\right]}^{T}$, $\cos(\psi_{i\_step})$ and $\sin(\psi_{i\_step})$ are derived from Equation (18), $q_{i\_step}={\left[\begin{array}{cccc} q_{0}^{i\_step} & q_{1}^{i\_step} & q_{2}^{i\_step} & q_{3}^{i\_step} \end{array}\right]}^{T}$ is the rotation quaternion estimated by the first EKF at the acceleration peak point of the ith step.

Secondly, compute the a *priori* state estimation $L_{i}^{-}$ and related error covariance matrix $P_{i}^{-}$:

(36) $$L_{i}^{-}\;=\;{\hat{L}}_{i-1}\;+\;\tilde{f}\left(qd_{i}\right)$$

(37) $$P_{i}^{-}=P_{i-1}+{\tilde{F}}_{i}P_{qd_{i}}{{\tilde{F}}_{i}}^{T}$$

where ${\hat{L}}_{i-1}$ is the a *posteriori* location estimation at the (i−1)th step, ${\tilde{F}}_{i}=\partial \tilde{f}/\partial qd$ is the Jacobian matrix computed at $qd=qd_{i}$, $P_{i-1}$ is the a *posteriori* error covariance matrix at the (i−1)th step:

(38) $$P_{qd_{i}}=\left[\begin{array}{cc} P_{q}^{i\_step} & 0\\ 0 & \sigma_{SL}^{2} \end{array}\right]$$

where $P_{q}^{i\_step}$ is the a *posteriori* error covariance matrix of $q_{i\_step}$, which can be obtained from Equation (33) of the first EKF; and $\sigma_{SL}^{2}$ is the error covariance of the step length estimation.

Thirdly, we combine KDE-based WiFi positioning results with observed landmarks to construct the measurement model:

(39) $$L_{i}^{observed}\;=\;\left[\begin{array}{l} L_{i}^{r}\\ L_{landmark} \end{array}\right]=L_{i}+{\tilde{v}}_{i}=L_{i}+\left[\begin{array}{l} {\tilde{v}}_{i}^{r}\\ {\tilde{v}}_{i}^{landmark} \end{array}\right]$$

where $L_{i}^{r}$ is the KDE-based WiFi positioning result using RSS vector collected at the ith step $r_{i\_step}$. Similar to Equations (9) and (10):

(40) $$L_{i}^{r}=\sum_{i=1}^{N_{i\_step}}{\tilde{w}}_{i}\left(r_{i\_step}\right)l_{i}$$

(41) $${\tilde{R}}_{i}^{r}={\tilde{v}}_{i}^{r}{\left({\tilde{v}}_{i}^{r}\right)}^{T}=\sum_{i=1}^{N_{i\_step}}{\tilde{w}}_{i}\left(r_{i\_step}\right)\left(\Sigma_{l}+\left(l_{i}-L_{i}^{r}\right){\left(l_{i}-L_{i}^{r}\right)}^{T}\right)$$

where ${\tilde{R}}_{i}^{r}$ is the measurement noise covariance matrix of WiFi positioning, $\left({\bar{r}}_{i},l_{i}\right)\in S_{i\_step}$ is the training pairs included in the trusted area of the ith step derived from Equation (11), $N_{i\_step}$ is the number of calibration points defined by the related trusted area:

(42) $${\tilde{R}}_{i}^{landmark}={\tilde{v}}_{i}^{landmark}{\left({\tilde{v}}_{i}^{landmark}\right)}^{T}=\left[\begin{array}{cc} \sigma_{x}^{2} & 0\\ 0 & \sigma_{y}^{2} \end{array}\right]$$

(43) $${\tilde{R}}_{i}={\tilde{v}}_{i}{\left({\tilde{v}}_{i}\right)}^{T}=[\begin{array}{cc} {\tilde{R}}_{i}^{r} & 0\\ 0 & {\tilde{R}}_{i}^{landmark} \end{array}]$$

where ${\tilde{R}}_{i}^{landmark}$ is the measurement noise covariance matrix of landmarks, which can be set according to the style of a landmark and related realistic environments.

Similarly to [28], the measurement noise of each landmark is set during the offline phase. Firstly, define an area covering all possible locations for a specific landmark. For example, when the pedestrian takes an elevator, the area inside the elevator includes all possible locations. Secondly, for simplicity, the measurement noise is assumed to follow two-dimensional Gaussian distribution. We choose a three-sigma ellipsoid to describe the covered area, where the pedestrian lying in the three-sigma ellipsoid can reach up as high as 98.89%. Thirdly, the center point of the defined area is set as the location of the landmark and the radius of the area is set to three-sigma. For example, the radius of an elevator is set as the half of the larger diagonal line of the rectangle. Finally, the standard deviation of the measurement noise is set as a third of the radius. The parameter setting may change according to different shapes of the landmarks and related environments. The influence of different settings will be studied in the future.

Finally, the a *posteriori* location estimation at the ith step ${\hat{L}}_{i}$ and covariance matrix $P_{i}$ are given:

(44) $$K_{i}=P_{i}^{-}{\left(P_{i}^{-}+{\tilde{R}}_{i}\right)}^{-1}$$

(45) $${\hat{L}}_{i}=L_{i}^{-}+K_{i}\left(L_{i}^{r}-L_{i}^{-}\right)$$

(46) $$P_{i}=P_{i}^{-}-K_{i}P_{i}^{-}$$

The computational cost of the proposed EKF-based fusion approach is comparable with that of the Kalman filter-based approach [12], while it is much less than that of the particle filter-based [14] approach. In our experimental environments as seen in Section 4.1, we obtain the averaged calculation time per walking step of twenty traces by running the approaches in Matlab. The averaged calculation time per walking step of the proposed fusion approach takes 0.166 s, including 0.115 s for the first EKF and 0.051 s for the second EKF. For the particle filter based approach with 1000 particles, it takes 10.4 s and, thus, unsuitable for running on a resource-limited smartphone.

## 4. Evaluation

### 4.1. Experimental Setup

**Figure 4:**
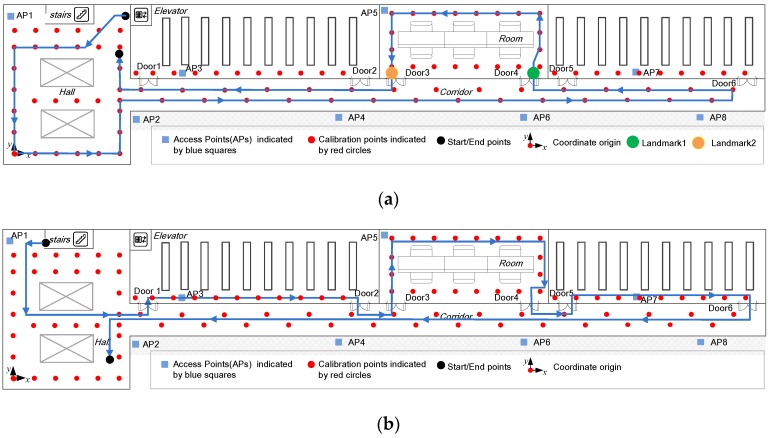
Experimental environment and the true walking path: (**a**) path one; (**b**) path two.

Experiments were performed in a typical indoor office area at the building of our department, whose size are 43.5 m × 11.2 m, as seen in Figure 4. We deploy a Samsung Galaxy S4 smartphone with the Android 4.2 operating system as the device involved in the experiments. The environment coordinate system is defined as seen in the lower-left of the hall, where the coordinate origin, *x*-axis, and *y*-axis are specified. For WiFi positioning, we construct the radio map by collecting RSS values at calibration points from eight APs distributed in the office environment during offline phase. All eight APs are hung in a relatively high place to reduce the signal attenuations caused by obstacles. The calibration points in the corridor are set two rows, while the distance between adjacent points along *x*-axis is the same as that in the hall. This setting is beneficial for improving the WiFi positioning accuracy in the corridor, especially along the *y*-axis, while requiring the same number of calibration points. Handheld device collected 100 RSS sample vectors per calibration point along four directions: *x*-axis, *y*-axis, and their opposite directions, each direction corresponding a quarter of the total number of samples. It should be noted that, the number of calibration points and their deployment may affect the WiFi positioning accuracy greatly. If the number of calibration points reduces or the walking path is far away from the calibration points, the WiFi positioning accuracy may deteriorate greatly [36] and even degrade the accuracy performance of the proposed fusion approach. During the online phase, real-time RSS vectors, gyroscope, accelerometer, and magnetometer data at a sampling rate of 2 Hz, 100 Hz, 50 Hz, and 10 Hz were collected, respectively.

The participant walks along the true path one and path two (indicated by the blue solid line) at least ten times, as seen in Figure 4a,b, respectively. The initial location and orientation of each experimental run are assumed to be known as a *priori* by exploiting the elevator and stair landmarks. At the beginning of each experimental run, the gyroscope is already well calibrated by the zero attitude update [35] method. We compare the proposed fusion approach with PDR using gyroscopes only, PDR combining gyroscopes with accelerometers, magnetometers, and landmarks, and WiFi positioning approaches. To validate the effectiveness of the KDE-based measurement model, we also compare the fusion approach whose measurement noise covariance matrix of WiFi positioning is set empirically [12], while the other modules of the fusion approach are the same as the proposed fusion approach. In our experiments, the measurement noise covariance matrix of WiFi positioning for the empirical fusion approach is set as a constant one, which makes the empirical fusion approach performs best for the testing path. To label the ground truth of the tracking trajectory and each walking step accurately, we exploit the square floor tiles and use a video to record the entire process of each walking path. Experiments for each path are carried out individually, and the positioning performance are the statistical results of the ten repeated tests for both two paths. The positioning error is defined as the Euclidean distance between estimated coordinate vectors and the true ones along the walking trajectory.

### 4.2. Performance Analysis

**Figure 5:**
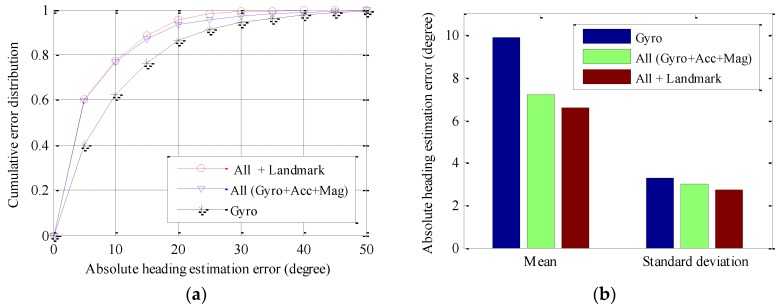
Performance comparisons of absolute heading estimation error of path one: (**a**) cumulative error distribution; and (**b**) mean and standard deviation.

**Figure 6:**
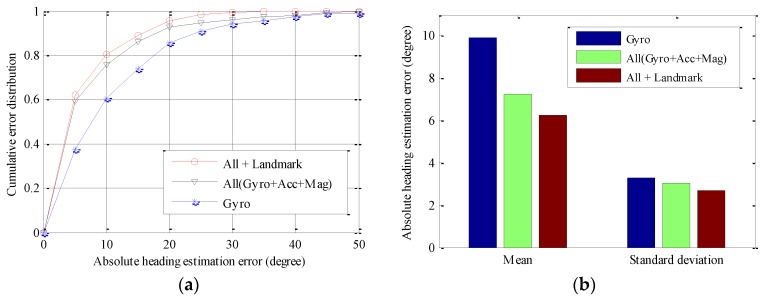
Performance comparisons of absolute heading estimation error of path two: (**a**) cumulative error distribution; and (**b**) mean and standard deviation.

Firstly, we evaluate the user heading estimation accuracy of various PDR-based approaches, since heading estimation bias is a main source of tracking error. Figure 5 compares user heading estimation accuracy of PDR using only a gyroscope (Gyro), using all sensors including gyroscopes, accelerometers, and magnetometers (All (Gyro + Acc + Mag)), and combining all sensors with landmarks (All + Landmark). As seen in Figure 5a, the All (Gyro + Acc + Mag) approach performs significantly better than that of the Gyro approach. This is because the former approach may fuse both acceleration and magnetic field signals, which contain tilt angle and yaw angle information of the device, respectively. For the All + Landmark approach, since the landmarks including passing through doors 2 and 3 are exploited to recalibrate the heading estimation, it may further improve heading estimation performance. As a result, the probability of large heading estimation errors reduces significantly. As seen in Figure 5b, mean absolute heading estimation error of the All + Landmark and the All (Gyro + Acc + Mag) approach reduce by 27.1% and 33.2% than that of the Gyro approach, respectively. Similarly, heading estimation accuracy improvement may also be obtained by combining all sensors with landmarks for the path two, as seen in Figure 6. The only difference is that, compared with All (Gyro + Acc + Mag) approach, the All + Landmark approach may obtain more significant heading estimation accuracy improvement, since more landmarks will be exploited to recalibrate the heading estimation.

**Figure 7:**
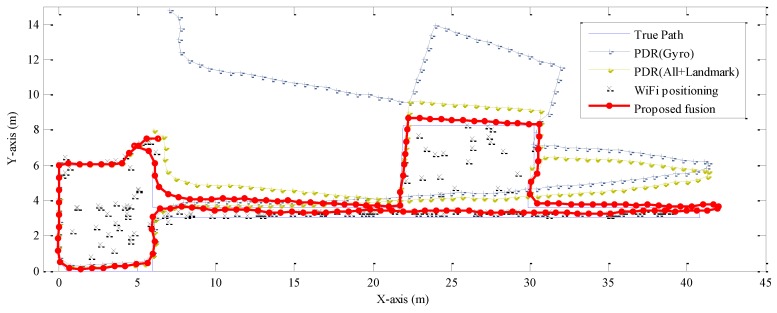
Comparisons between the true path one, trajectories of the proposed fusion approach, PDR (Gyro), PDR (All + Landmark), and WiFi positioning.

Secondly, we compare pedestrian navigation performance along path one of the proposed fusion approach, PDR (Gyro), PDR (All + Landmark), and WiFi positioning. As seen in Figure 7, PDR-based approaches have comparable tracking accuracy with the proposed fusion approach along the initial walking path in the hall, while performing progressively worse with the heading estimation error accumulated. Tracking performance of PDR (All + Landmark) is significantly better than that of PDR (Gyro), since the heading estimation error of the former approach is smaller than that of the latter one. Particularly, when a pedestrian passes through Doors 4 and 3, the related landmark 1 and landmark 2 are identified and exploited to recalibrate the positioning and heading estimation results. Therefore, tracking errors of PDR (All + Landmark) reduce significantly immediately after passing through Doors 4 and 3.

**Figure 8:**
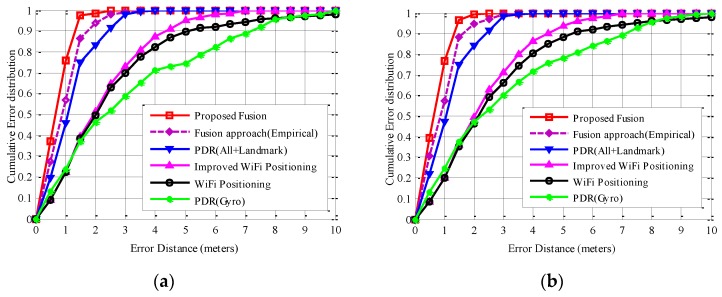
Accuracy comparisons between the proposed fusion approach, fusion approach (Empirical), PDR (All + Landmark), PDR (Gyro), WiFi positioning, and the improved WiFi positioning: (**a**) path one; (**b**) path two.

Though individual WiFi positioning obtains relatively inaccurate tracking results along the whole walking path, the tracking error does not accumulate as PDR approach. By deploying the trusted area of WiFi positioning and detection of outlier RSS measurements, probability of large positioning error reduces significantly, as seen in Figure 8. WiFi positioning also provides useful localization information. For example, when the pedestrian walks along the corridor, the *y*-axis coordinates of the WiFi positioning results are always accurate and restricted into a small region, since the matched calibration points are almost all in the corridor. Moreover, by deploying the KDE-based model, uncertainty of WiFi positioning results is measured accurately, thus rendering WiFi positioning results with a larger error gain a smaller weight during the fusion process of EKF. Therefore, the proposed fusion approach performs best, due to an effective correction of PDR by fusing WiFi positioning and landmarks.

Finally, we compare positioning accuracy performance between the proposed fusion approach and the other approaches. Figure 8 shows the corresponding cumulative error distributions of compared approaches of both paths. Clearly, the proposed fusion approach obtains substantial accuracy improvement than compared individual approaches. As seen in Figure 8a for path one, particularly, accuracy within 1 m of the proposed fusion approach is 75.8%, while those of the fusion approach (empirical), PDR (All + Landmark), PDR (Gyro), and WiFi positioning are 56.9%, 45.8%, 23.9%, and 22.2%, respectively. Accuracy within 2 m of the proposed fusion approach is 98.2%, while those of the fusion approach (empirical), PDR (All + Landmark), PDR (Gyro), and WiFi positioning are 93.7%, 83.0%, 46.4%, and 49.7%, respectively. Accuracy within 6 m of the proposed improved WiFi positioning approach is 97.8%, while that of the original KDE-based WiFi positioning approach is only 92.2%. This is because most of large WiFi positioning errors are excluded by restricting the matched calibration points within a trusted area and identifying outlier RSS measurements.

Compared with the proposed fusion approach, the fusion approach (empirical) obtains significant accuracy performance degradation, because the measurement noise of WiFi positioning is set empirically and the uncertainty of WiFi positioning results cannot be handled. For example, when real-time RSS samples are corrupted in the complex propagation environment, the fusion approach (empirical) empirically setting the measurement noise may even bias the fusion results by giving WiFi positioning results of a large error a large weight during the fusion process. This case can be avoided effectively by the proposed fusion approach, whose measurement noise is adaptive tuned by the KDE-based method. PDR (Gyro) approach performs the worst, since the tracking error progressively increases with the accumulated heading estimation errors and positioning errors. Similar conclusions can also be derived from Figure 8b for path two. Additionally, all approaches involving PDR along path two perform slightly better than those of corresponding approaches along path one, since there are more landmarks exploited to calibrate the location estimation and heading estimation.

Table 3 shows the performance comparisons of various positioning error (m) measures. For simplicity, we give statistical results of two paths, since the performance of two paths is very close. Compared with the Fusion Approach (Empirical), PDR (All + Landmark), PDR (Gyro), WiFi positioning, and our proposed improved WiFi positioning, combining a trusted area with outlier detection, the proposed fusion approach reduces mean positioning errors by 25.0% (0.24 m), 41.0% (0.50 m), 76.9% (2.40 m), 76.1% (2.29 m), and 70.4% (1.71 m), while it reduces standard deviations by 24.1% (0.14 m), 40.5% (0.30 m), 83.3% (2.19 m), 83.6% (2.25 m), and 77.4% (1.51 m), respectively.

In summary, the proposed fusion approach obtains significant positioning accuracy improvement than various individual approaches and the fusion approach (empirical) for the following three reasons. Firstly, heading estimation performance of PDR is improved by fusing gyroscopes, accelerometers, and magnetometers, and recalibrated by exploiting landmarks. Secondly, measurement model parameters of WiFi positioning are adaptively tuned by the KDE-based method and, thus, WiFi positioning results are fused to improve the positioning accuracy more effectively. Thirdly, various landmarks are identified by a decision tree approach and exploited to recalibrate the location estimation.

## 5. Conclusions

In this paper, we propose an extended Kalman filter-based positioning approach by fusing WiFi, PDR, and landmarks. For WiFi positioning, instead of setting measurement noise parameters manually and empirically, we deploy a kernel density estimation model to measure it accurately and adaptively. Furthermore, by deploying a trusted area of WiFi positioning to restrict matched calibration points and detecting outlier RSS measurements, probability of WiFi positioning results with a large error distance reduces significantly. For user heading estimation of PDR, we develop another EKF model to integrate gyroscopes, accelerometers, magnetometers, and landmarks. Landmarks including elevators, escalators, stairs, and doors are identified by a decision tree approach. Then, they are exploited to recalibrate both user heading estimations and positioning results. Experimental results show that, compared with the fusion approach empirically setting measurement noise parameters of WiFi positioning, PDR using gyroscopes, accelerometers, magnetometers, and landmarks, WiFi positioning and PDR using a gyroscope alone, the proposed fusion approach reduces mean positioning errors by 25.0% (0.24 m), 41.0% (0.50 m), 76.1% (2.29 m), and 76.9% (2.40 m), respectively.

In future works, we will further consider the situation when orientation and carrying position of a smartphone are both free, such as placed in a pocket, which has been paid few attentions. Additionally, the proposed fusion approach will be tested in a larger and more complicated indoor environment including multi-floors and escalators.

## Figures and Tables

**Table 1:** Key symbols used in the proposed fusion approach.

Notations	Definitions
$l_{i}$	Coordinate vector of the *i*-th calibration point
$L_{i}$	Coordinate vector of the *i*-th walking step
${\hat{L}}_{i}$	Ultimate location estimation result at the *i*-th step
$L_{i}^{r}$	WiFi positioning result using RSS vector collected at the *i*-th step
${\bar{r}}_{i}$	Mean RSS vector at the *i*-th calibration point
$℘\left(r;{\bar{r}}_{i},\Sigma_{r}\right)$	Gaussian kernel function with mean vector ${\bar{r}}_{i}$ and covariance matrix $\Sigma_{r}$
d	Number of APs used in positioning
DCS/UCS/GCS	Device/User/Global Coordinate System
$SL_{i}$	Step length of the *i*-th walking step
$\psi_{i\_step}$	Yaw angle (user heading) estimated at the *i*-th step at GCS
$C_{GCS}^{DCS}$	Direction cosine matrix between DCS and GCS
$q_{k}$	Rotation quaternion vector at time instants $kT_{s}$

**Table 2:** Confusion matrix for landmarks identification.

Motion States	Stairs	Elevators	Escalators	Walking	Standing	Doors
Stairs	0.995	0	0	0.005	0	0
Elevators	0	1.0	0	0	0	0
Escalators	0	0	1.0	0	0	0
Walking	0	0	0	1.0	0	0
Standing	0	0	0	0	1.0	0
Doors	0	0	0	0.02	0	0.98

**Table 3:** Performance comparisons of various positioning error (m) measures.

Compared Approach	Proposed Fusion	Fusion Approach (Empirical)	PDR (All + Landmark)	PDR (Gyro)	WiFi Positioning	Improved WiFi Positioning
Mean error	**0.72**	0.96	1.22	3.12	3.01	2.43
Standard deviation	**0.44**	0.58	0.74	2.63	2.69	1.95
Medium error	**0.68**	0.89	1.08	2.30	2.12	2.03
75 percentile	**0.92**	1.32	1.55	4.66	3.44	3.21
90 percentile	**1.35**	1.73	2.10	7.05	5.26	4.42
